# 3D Evaluation of the Lamina Cribrosa with Swept-Source Optical Coherence Tomography in Normal Tension Glaucoma

**DOI:** 10.1371/journal.pone.0122347

**Published:** 2015-04-15

**Authors:** Kazuko Omodaka, Takaaki Horii, Seri Takahashi, Tsutomu Kikawa, Akiko Matsumoto, Yukihiro Shiga, Kazuichi Maruyama, Tetsuya Yuasa, Masahiro Akiba, Toru Nakazawa

**Affiliations:** 1 Department of Ophthalmology, Tohoku University Graduate School of Medicine, Sendai, Japan; 2 Graduate School of Science and Engineering, Yamagata University, Yamagata, Japan; 3 Topcon Corporation, Tokyo, Japan; 4 Department of Retinal Disease Control, Ophthalmology, Tohoku University Graduate School of Medicine, Sendai, Japan; 5 Department of Advanced Ophthalmic Medicine, Tohoku University Graduate School of Medicine, Sendai, Japan; Universidade Federal do Rio de Janeiro, BRAZIL

## Abstract

**Purpose:**

Although the lamina cribrosa (LC) is the primary site of axonal damage in glaucoma, adequate methods to image and measure it are currently lacking. Here, we describe a noninvasive, *in vivo* method of evaluating the LC, based on swept-source optical coherence tomography (SS-OCT), and determine this method’s ability to quantify LC thickness.

**Methods:**

This study comprised 54 eyes, including normal (n = 18), preperimetric glaucoma (PPG; n = 18), and normal tension glaucoma (NTG; n = 18) eyes. We used SS-OCT to obtain 3 x 3 mm cube scans of an area centered on the optic disc, and then synchronized reconstructed B- and *en-face* images from this data. We identified the LC in these B-scan images by marking the visible borders of the LC pores. We marked points on the anterior and posterior borders of the LC in 12 B-scan images in order to create a skeleton model of the LC. Finally, we used B-spline interpolation to form a 3D model of the LC, including only reliably measured scan areas. We calculated the average LC thickness (avgLCT) in this model and used Spearman's rank correlation coefficient to compare it with circumpapillary retinal nerve fiber layer thickness (cpRNFLT).

**Results:**

We found that the correlation coefficient of avgLCT and cpRNFLT was 0.64 (p < 0.01). The coefficient of variation for avgLCT was 5.1%. AvgLCT differed significantly in the groups (normal: 282.6 ± 20.6 μm, PPG: 261.4 ± 15.8 μm, NTG: 232.6 ± 33.3 μm). The normal, PPG and NTG groups did not significantly differ in age, sex, refractive error or intraocular pressure (IOP), although the normal and NTG groups differed significantly in cpRNFLT and Humphrey field analyzer measurements of mean deviation.

**Conclusion:**

Thus, our results indicate that the parameters of our newly developed method of measuring LC thickness with SS-OCT may provide useful and important data for glaucoma diagnosis and research.

## Introduction

Glaucoma is a group of progressive optic neuropathies in which the retinal ganglion cells and their axons slowly degenerate, a process that results in a corresponding pattern of visual field loss [1]. Glaucoma affects over 70 million people worldwide, and has become the second most common cause of blindness [2,3]. High intraocular pressure (IOP) is the most well-known and important risk factors, but glaucoma is believed to be a multifactorial disease [4–6]. Past studies showed that glaucomatous axonal degeneration is closely related to morphological changes in the lamina cribrosa (LC), a structure located in the bottom of the optic nerve cup that is composed of a series of porous collagenous plates [7–10]. Retinal ganglion cell (RGC) axons and the central retinal vessels pass through pores in the LC. Glaucomatous deformation of the LC compresses these axons, preventing axoplasmic flow [11] and causing RGC apoptosis [12–14]. Thus, the LC is presumed to be the primary site of glaucomatous axonal damage and is a promising subject of ophthalmological research. Improved methods to image the LC *in vivo* would therefore be a great benefit.

Optical coherence tomography (OCT), a technology introduced by Huang et al. [15], is well established as a way to measure circumpapillary retinal nerve fiber layer thickness (cpRNFLT) in early glaucoma diagnosis [16–18]. Recent innovations in OCT, such as new segmentation algorithms and the development of spectral-domain (SD)-OCT have increased the usefulness of OCT instruments. Modern versions of OCT allow the visualization of each retinal layer in the macula, including the ganglion cell complex (GCC), which is the layer between the RNFL and the inner plexiform layer (IPL), and the ganglion cell layer plus IPL (GCL+IPL) [19]. Studies of these individual layers [20,21] have shown that measurements of macular GCC thickness, in particular, are useful, having the same value for glaucoma diagnosis as measurements of overall cpRNFLT. Thus, OCT evaluation has become an essential part of objective examinations for glaucoma.

The most recently introduced OCT techniques have greatly improved imaging of the deeper parts of the retina [22]. Enhanced depth imaging (EDI), based on SD-OCT, has allowed glaucoma specialists to visualize the anterior surface of the LC [23,24], measure the thickness of the LC [25,26], and evaluate the LC in three dimensions (3D) [27]. Swept-source OCT (SS-OCT), which uses a high-penetration, long-central-wavelength laser, has also significantly improved observation of the LC in glaucoma [28]. Technological progress has therefore enabled new methods to examine the LC in great detail. Nevertheless, even with these new techniques, it is still difficult to confidently identify the posterior border of the LC in OCT images.

In this study, we report on newly developed software that enables evaluation of the LC in 3D and facilitates the manual determination of LC thickness. This software reconstructed 12 radial B-scan OCT images of the optic nerve head from a set of 256 horizontal 3D OCT B-scan images, thus enabling the simultaneous visualization of both B-scan images and *en-face* images of the optic nerve, as well as the motion synchronization of these images. In this study, we identified the LC in *en-face* images of the optic nerve according to the visibility of the pores of the LC structure, i.e., the anterior border was defined as the point where the pores became visible in the images and the posterior borders was defined as the point where the pores ceased to be visible. After thus identifying the borders of the LC, we calculated parameters of the thickness of the LC. Finally, we assessed the diagnostic usefulness of these parameters by comparing them with the patients’ visual fields and cpRNFLT. Thus, this study evaluated the ability of new measurement parameters of the LC, obtained with SS-OCT and new software, to usefully measure glaucomatous change in the LC in patients.

## Methods

### Inclusion Criteria

A total of 54 eyes of 54 Japanese adult subjects were included in this retrospective, cross-sectional study. The study population included normal subjects (n = 18), preperimetric glaucoma patients, (PPG, n = 18) and patients with normal tension glaucoma (NTG, n = 18).

We recorded baseline clinical parameters for each patient, including age, sex, and refractive error. We measured baseline best-corrected visual acuity (BCVA) with a standard Japanese decimal visual acuity chart, and converted it to the logarithm of the minimum angle of resolution (logMAR) for statistical analysis. IOP was measured with Goldmann applanation tonometry at the time of the initial diagnosis of NTG, before the use of any medication for the disease. Before pupil dilation, slit-lamp biomicroscopy and gonioscopy were performed. Central corneal thickness was measured with anterior-segment OCT (CASIA, Tomey Corporation, Nagoya, Japan). Following pupil dilation with tropicamid (midrin M, Santen pharmaceutical, Osaka, Japan), stereoscopic examination of the ONH was performed, fundus and disc photographs (the latter in color) were taken and ocular biometry (IOL Master; Carl Zeiss Meditec) was performed with full pupil dilation. This study adhered to the tenets of the Declaration of Helsinki, and the protocols were approved by the Clinical Research Ethics Committee of the Tohoku University Graduate School of Medicine (study 2012-1-574). All subjects provided written informed consent for their participation in this study.

The normal subjects were free of ocular disease. Inclusion criteria for the subjects with NTG were: (1) diagnosis of NTG; (2) a spherical equivalent refractive error of > -5.00 diopters (D); and (3) an abnormal glaucomatous optic disc (with diffuse or focal thinning of the neuroretinal rim) and an abnormal visual field consistent with glaucoma, defined according to the Anderson-Patella criteria [29]. Inclusion criteria for the subjects with PPG were: (1) clinical features that led the diagnostician to suspect glaucoma, i.e., an optic disc with a glaucomatous appearance, such as one with neuroretinal rim narrowing, cupping or suspicious RNFL defects and (2) a normal visual field, defined as a pattern standard deviation within a 95% confidence limit and results from a glaucoma hemifield test within the normal limits. All eyes in this study had a BCVA of ≥ 20/40. Patients were excluded if any of the following arose during follow-up: (1) ocular disease other than NTG; (2) systemic disease affecting the visual field; (3) intraocular surgery; or (4) cataract progression.

### Measurement of the lamina cribrosa with swept-source optical coherence tomography

Scanning of the LC with swept-source OCT (DRI OCT-1; Topcon Corp., Tokyo, Japan) was performed at Tohoku University Hospital in Japan. The DRI OCT-1 system uses an axial scan rate of 100 kHz and operates in the 1-μm wavelength region. This yields images with an 8-μm axial resolution in tissue. The transverse resolution of the device was set to approximately 20 μm. A single OCT image, consisting of 1000 A-lines, was acquired in 10 msec. The imaging depth was 2.6 mm in the tissue, and the lateral scan length was adjustable. These features made DRI OCT-1 a suitable instrument for visualization of deep ocular tissue, including the LC and choroid.

### Creation of pseudo-radial images and ***en-face*** images

**Fig 1:**
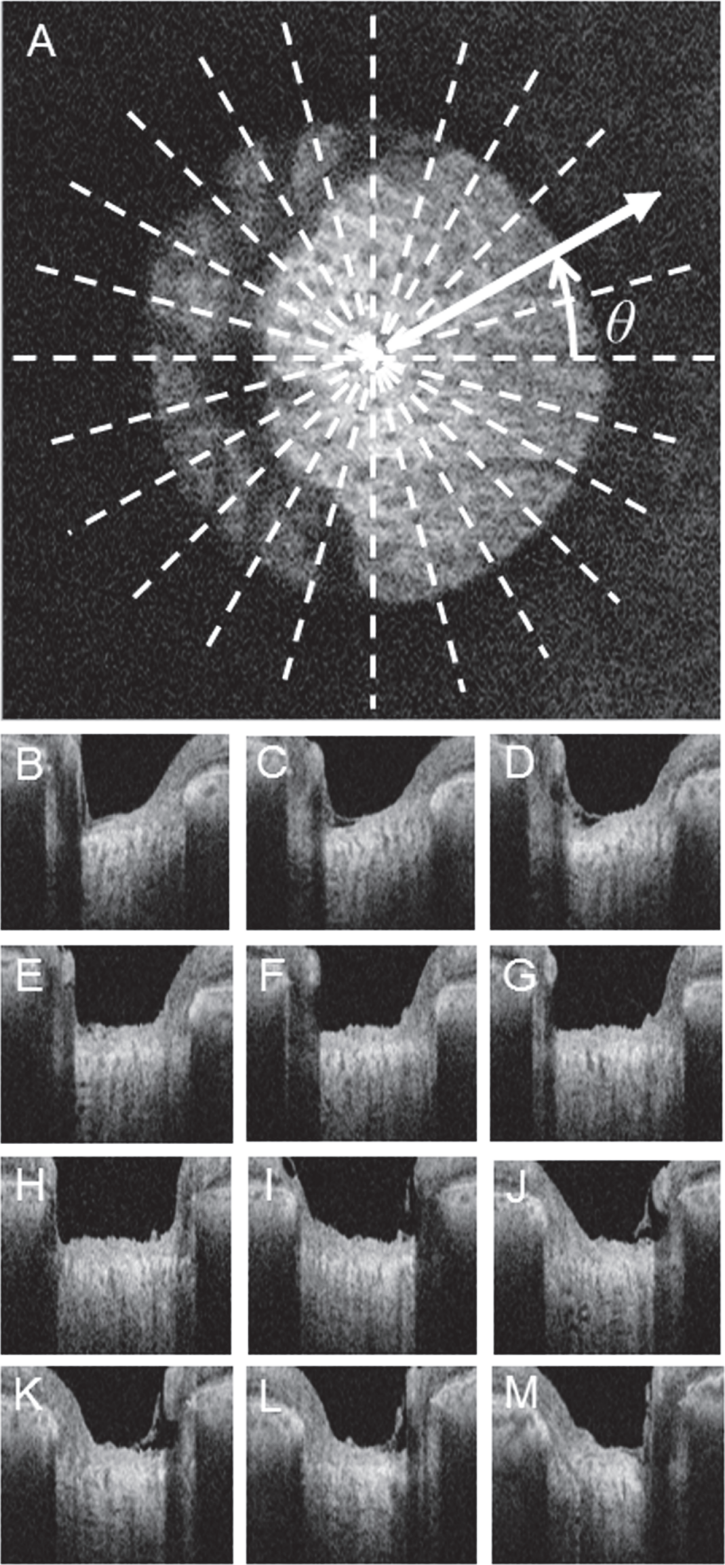
Pseudo *en-face* image and 12 pseudo-radial B scan images. (A) Reconstructed *en-face* image. (B-M) Reconstructed B-scan images. *r*, *θ* polar coordinate system on the *xy*-plane, where white lines denote the intersections of *θ* pseudo-radial images.

We used the (*r*, *θ*) polar coordinate system in the plane parallel to the *xy*-plane, with Bruch’s membrane opening (BMO) as the origin, as shown in Fig 1A, where *θ* is the angle between the *r*- and *x*-axes. For each value of *θ*, we referred to a cross-section that included the corresponding *r*-axis and was parallel to the *z*-axis, and created a *θ* pseudo-radial image. By changing the value from 0 to π in steps of Δ*θ*, we generated π/Δ*θ* pseudo-radial images from a single 3D-OCT image. In this study, we generated 12 pseudo-radial images with *θ* = 15 degrees (Fig 1B and 1M).

We also created a set of *en-face* images from each 3D-OCT image. In order to reduce speckle noise, a motion-averaging technique was applied to the 3D-OCT images in the *z* direction. Five images, comprising 1 *en-face* image at a *z* coordinate of interest and 4 *en-face* images from above and below it, were used for this averaging process.

### Identification of the upper and lower borders of the LC and the flank of the LC

**Fig 2:**
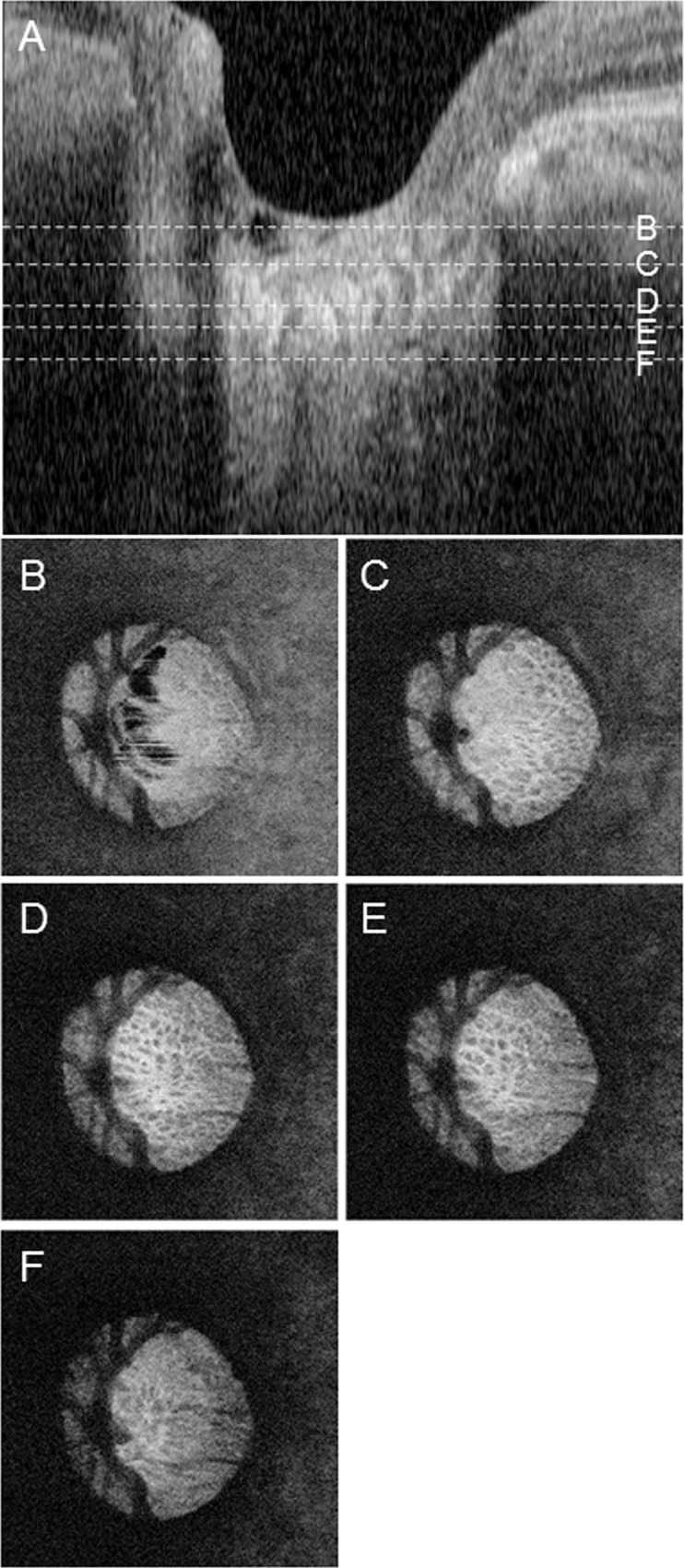
Correlation between B-scan and *en-face* images. (A) B-scan image with dotted lines showing the position of the *en-face* images below. (B) Upper area of the lamina cribrosa (LC). (C) Upper border of the LC. (D) Centerline of the LC. (E) Lower border of the LC. (F) Lower area of the LC.

**Fig 3:**
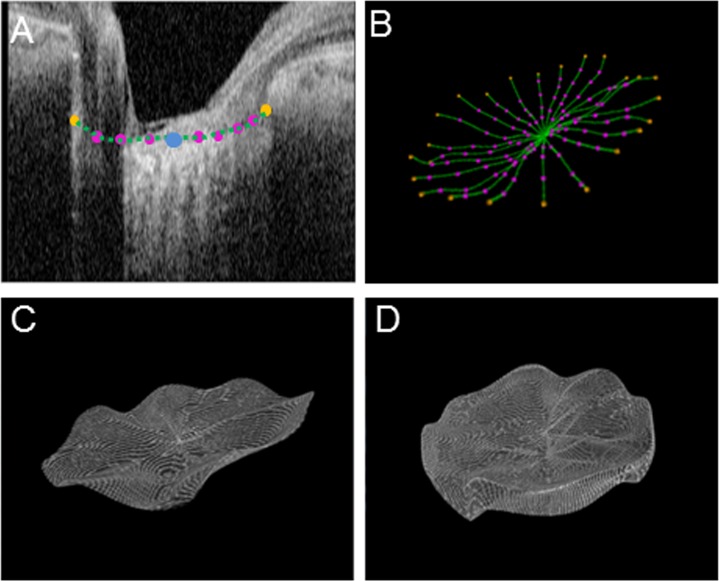
3D modeling of the lamina cribrosa (LC). (A) Plotted points on a pseudo-radial image and the results of B-spline interpolation, where the blue dot is the center of Bruch’s membrane opening, the orange dots are the terminal points, and the pink dots are other plotted points. (B) Wireframe model of the upper surface of the LC, where the orange dots are terminal points and the pink dots are other plotted points. (C) B-spline interpolated curved surface of the upper side of the LC. (D) 3D model of the LC.

The upper and lower borders of the LC and the flank of the LC were all identified with a similar method, and the following description of the technique to identify the upper border of the LC can therefore be considered representative (Fig 2). For each *θ* pseudo-radial image, we chose a coordinate on the corresponding *r*-axis. Then, we moved the *en-face* image in the *z* direction. We identified the upper border of the LC at the chosen *r*-coordinate and recorded the corresponding *z*-coordinate of the upper border of the LC. This was only done when we were able to discriminate the pores of the LC at the chosen *r*-coordinate in the *en-face* image. Visible pores were defined as small, round low-intensity areas surrounded by hyper-scattered fibrous tissue. Large vessel shadows were easily recognizable by their continuous structure in sequential *en-face* images. It was possible to effectively exclude small artifacts and vessel shadowing, as the accumulated pores were visible over a wider area than the small artifacts. Repeating this procedure while changing the *r*-coordinate, we acquired a set of data (*r*, *z*) characterizing a curve, which intersected the LC in the *θ* pseudo-radial image. Fig 3A shows an example of how the data were collected: the blue dot is the center of the BMO, the orange dots are the terminal points of the LC curve, and the pink dots are other manually plotted points. In this study, we collected at least 4 points, including the 2 terminal points, for the interpolation described in the next section, but we did not use a constant sampling interval on the *r*-axis. If the border of the LC had a complicated shape, such as a W-shape, the observer collected additional points in order to accurately measure the anterior and posterior borders of the LC. In most cases, more than 8 points were collected. Naturally, the accuracy of the interpolation could be increased by sampling *r*-coordinates more densely in regions where the curve rapidly varied. The above procedure was conducted for all *θ* pseudo-radial images.

### 3D modeling of the LC

Since the upper and lower borders of the LC and the flank of the LC were all modeled with a similar procedure, the following explanation of the technique to model the upper border of the LC can be considered representative. The modeling was performed first in the *r* direction and then in the *θ* direction using B-spline interpolation.

#### (a) *r*-directional interpolation

Using a data set acquired from a *θ* pseudo-radial image, we performed B-spline interpolation in the *r* direction, shown in Fig 3A as a green line, which represents the B-spline interpolated curve. After completing the interpolation for all values of *θ* we obtained a wireframe model radiating from the BMO origin, as shown in Fig 3B, where the pink dots show plotted points and the orange dots are terminal points.

#### (b) *θ* directional interpolation

Next, we conducted interpolation in the *θ* direction. Here, it should be noted that not only the number of sampling points, but also the sampling interval differed for each value of *θ*. Thus, we re-sampled the *r* coordinate. First, we used an index *i* (*i* = 0, 1, …, *M*-1) to differentiate the 2π/Δ*θ (= M) r*-axes generated by changing the value from 0 to 2π in steps of Δ*θ*. When the angle between the *r*-axis and the *x*-axis was *θ = i* Δ*θ*, we referred to it as the *r*_*i*_-axis, where coordinates on the *r*_*i*_-axis were non-negative and the origin of the *r*_*i*_-axis corresponded with the center of the BMO.

As the terminal point of the upper border of the LC on the *r*_*i*_-axis was known, we collected *N*+1 coordinates on the *r*_*i*_-axis, *r*_*i* 0_, *r*_*i* 1_, *r*_*i* 2_, …, *r*_*i N*_ by equally sectioning the *r*_*i*_-axis between the origin and the terminal point, where *r*_*i* 0_ and *r*_*i N*_ corresponded with the BMO origin and the terminal point, respectively. Although the z coordinates at *r*_*i j*_ (*j* = 1, 2, …, *N*-1) were unknown, the intersection of the upper border of the LC and the *r*_*i*_-axis were modeled as a smooth and continuous curved line in the *r*-directional interpolation. Therefore, we were able to obtain the z coordinates of the upper border of the LC at all *r*_*i j*_ from the interpolated function. Applying the procedure to all *r*_*i*_-axes, we obtained a data set of (*r*_*i j*_, *z*_*i j*_) (*i* = 0, 1, …, *M*-1, *j* = 1, 2, …, *N*), where *z*_*i j*_ was a z coordinate at *r*_*i j*_ obtained from the interpolated curve.

Then, we selected a data subset (*r*_*i j*_, *z*_*i j*_) (*i* = 0, 1, …, *M*-1) with *j* fixed from (*r*_*i j*_, *z*_*i j*_) (*i* = 0, 1, …, *M*-1, *j* = 1, 2, …, *N*). Connecting these points in ascending order with respect to *i* from 0 to *M* with (*r*_*M j*_, *z*_*M j*_) = (*r*_0 *j*_, *z*_0 *j*_), we were able to reconstruct a closed, smooth, and continuous 3D curve around the BMO origin. Applying this procedure to all values of *j*, we could obtain an interpolated curved border of the top of the LC if *N* was sufficiently large. Fig 3C shows the results of a B-spline interpolation with *M* = 24, *N* = 140.

Continuing the above procedure for the lower border and flank of the LC allowed us to create a 3D model of the LC (Fig 3D). In order to more easily carry out this analysis, we developed an interactive software system with a graphical user interface on a PC platform (CPU: Intel 1.8GHz Core i7-4500U, memory: 8 GB, OS: Windows 7) using the computer language C#.

### Establishment of reliably measurable region

**Fig 4:**
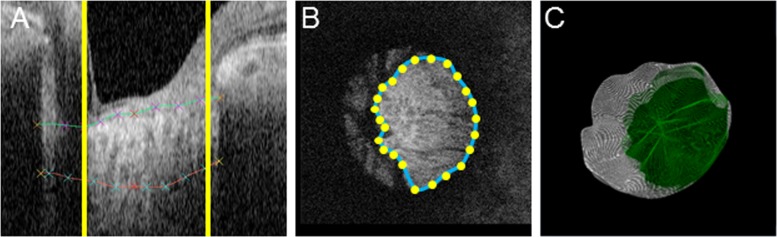
Establishment of reliably measurable region. (A) Reliably measurable region in a pseudo-radial B-scan image. The yellow vertical lines show the border of the reliably measurable region. (B) The reliably measurable region in the *xy*-plane, where the yellow dots show reliable points. (C) 3D model of the LC with the reliably measurable region shown in green.

Previous efforts to create 3D models of the LC have been negatively affected by the shadows cast by blood vessels in OCT scans, which can prevent the definitive identification of the LC in certain areas. In order to circumvent this problem, which can reduce the accuracy of measurements of LC thickness, we set out to isolate the area of the LC that could be reliably measured. For this purpose, we added a post-processing function to our software that efficiently selected the reliably measurable region of the scan. This region was chosen as follows: first, the user manually marked the reliably measurable region in each *θ* pseudo-radial image, shown in Fig 4A as the region between the yellow vertical lines, with the green and red curves showing the upper and lower borders of the LC, respectively. The appearance from a top view is shown in Fig 4B, where the yellow dots represent intersections of the yellow vertical lines in Fig 4A, i.e., the boundaries of the reliably measurable region in the *θ* pseudo-radial image and the *en-face* plane. Next, the software produced a reconstructed closed curve by connecting the dots using B-spline interpolation with a periodic boundary condition (shown as the blue curved line in Fig 4B). Finally, supposing a solid body with the cross-section chosen with this process extended in the *z* direction, the software determined the reliably measurable region in 3D, as the intersection of the established 3D model of the LC and the solid body. Fig 4C shows an example of a 3D model with the reliably measurable region, as determined with B-spline interpolation. In the figures, the green surface inside the 3D model of the LC represents the boundary of the reliably measurable region. Finally, the average thickness of the 3D LC model was calculated.

### Reproducibility of average thickness of the LC

To assess the intrasession reproducibility of our measurements of the average thickness of the LC (avgLCT), we calculated the coefficient of variation (CV) of 3 separate calculations, all made by the same software operator analyzing the same set of volume scan data from the optic disc. We also determined the inter-observer agreement between two investigators.

### Visual field analysis and circumpapillary retinal nerve fiber layer

CpRNFLT was measured with 3D OCT-2000 (version 8.00; Topcon Corp.) Twelve circular scans (3.46 mm in diameter) centered on the optic disc were merged, and the resulting average cpRNFLT was used in the analysis. Mean deviation (MD) values were obtained with the Swedish interactive threshold algorithm (SITA)-standard strategy and the 24–2 program of the Humphrey field analyzer (HFA, Carl Zeiss Meditec, Dublin, California, USA). Only reliably measured values were used (<20% fixation errors, <33% false positive results, and <33% false negative results). All examinations were performed within a 1-month period.

### Statistical analysis

The Kruskal-Wallis test, followed by the Steel-Dwass test, was used to determine the significance of differences between the subject groups (Normal, PPG, and NTG). The significance level was set at *P* <0.05. Spearman's correlation analysis was used to determine the correlation between avgLCT and cpRNFLT, using JMP software (version 10.0.2, SAS Institute Japan Inc., Tokyo, Japan).

## Results

The demographic data of the patients in this study is summarized in Table 1. In the normal, PPG, and NTG groups, respectively, the average age was 59.8 ± 10.4 years, 61.1 ± 8.0 years and 65.8 ± 7.0 years, the average spherical equivalent refractive error was -0.89 ± 1.57 D, -0.12 ± 1.17 D and -1.04 ± 1.86 D, and the average IOP was 14.7 ± 2.9 mmHg, 14.6 ± 1.1 mmHg and 14.3 ± 2.6 mmHg. These data did not differ significantly between the groups. In the normal, PPG, and NTG groups, respectively, the average MD was 0.89 ± 0.66 dB, 0.84 ± 0.75 dB and -9.64 ± 6.21 dB and the average cpRNFLT was 104.0 ± 9.6 μm, 93.7 ± 7.9 μm and 73.8 ± 13.8 μm. There were no differences in age, sex, refractive error, or IOP in the normal, PPG, and NTG groups (Table 1). The normal and PPG groups differed significantly with the NTG group in MD and cpRNFLT, but the normal and PPG groups did not differ with each other. Furthermore, there were no difference of the ratio between the reliably measurable area and the Bruch’s membrane opening area in normal, PPG groups, and NTG groups (Table 2).

**Table 1:** Demographic data for this study.

	Normal (n = 18)	PPG (n = 18)	NTG (n = 18)	*P* value
Age (years)	59.8 ± 10.4	61.1 ± 8.0	65.8 ± 7.0	NS
Male: Female	10：8	12：6	7：11	NS
Refractive error (D)	-0.89 ± 1.57	-0.12 ± 1.17	-1.04 ± 1.86	NS
IOP (mmHg)	14.7 ± 2.9	14.6 ± 1.1	14.3 ± 2.6	NS
MD (dB)	0.89 ± 0.66	0.84 ± 0.75	-9.64 ± 6.21	<0.01*
cpRNFLT (μm)	104.0 ± 9.6	93.7 ± 7.9	73.8 ± 13.8	<0.01*

D: diopter. IOP: intraocular pressure. MD: mean Humphrey field analyzer deviation. cpRNFLT: circumpapillary nerve fiber layer thickness. PPG: preperimetric glaucoma. NTG: normal-tension glaucoma. P values were determined with the Kruskal-Wallis test for age, refractive error, IOP and cpRNFLT, and the Chi-square test for sex.

**Table 2:** Comparison of the ratio between the reliably measurable area and the Bruch’s membrane opening area in normal, preperimetric glaucoma, and normal tension glaucoma patients.

	Normal (n = 18)	PPG (n = 18)	NTG (n = 18)	*P* value
Reliably measurable area (mm^2^)	1.02 ± 0.24	1.07 ± 0.35	1.08 ± 0.37	NS
BMO area (mm^2^)	2.32 ± 0.41	2.45 ± 0.44	2.36 ± 0.54	NS
Ratio of reliably measurable area to BMO area (%)	44.6 ± 10.3	43.7 ± 11.8	45.9 ± 13.9	NS

BMO: Bruch’s membrane opening. PPG: preperimetric glaucoma. NTG: normal-tension glaucoma. P values were determined with the Kruskal-Wallis test.

**Fig 5:**
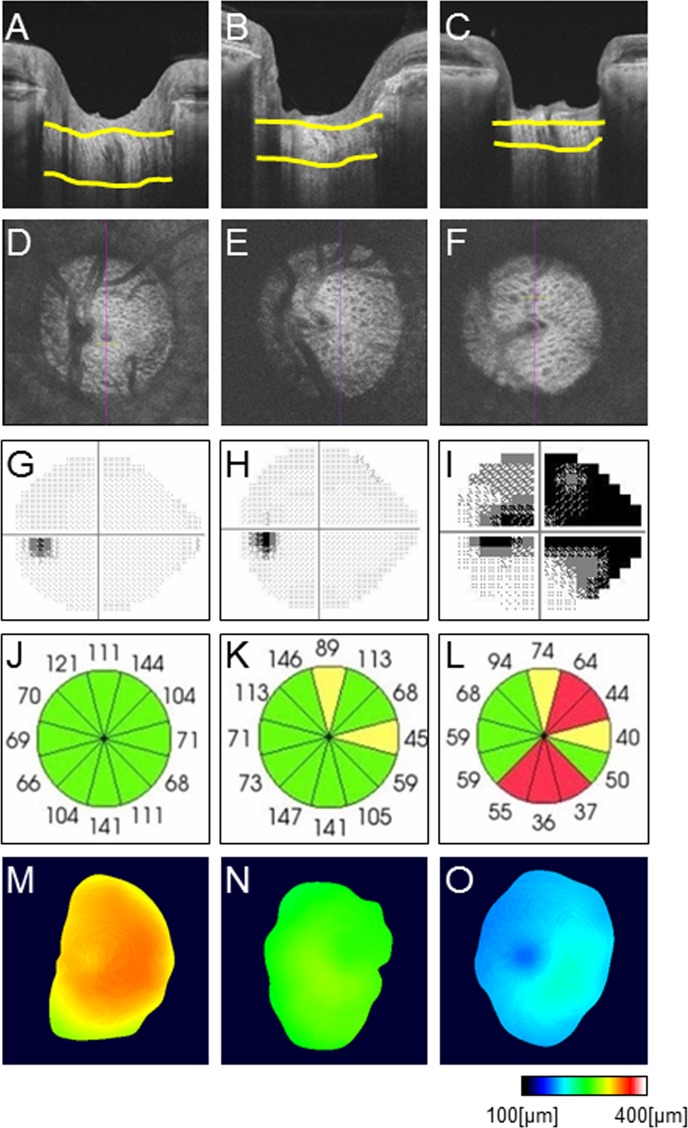
Representative B-scan images of normal eyes, preperimetric glaucoma eyes, and eyes with normal-tension glaucoma. (A-C) B-scan images. (D-F) *en-face* images. (G-I) Grayscale Humphrey field analyzer image. (J-L) 12 clock-wise sectors of OCT-measured circumpapillary retinal nerve fiber layer thickness. (M-O) Representative lamina cribrosa (LC) thickness map showing the reliably measurable area. (A, D, G, J, M) Normal. (B, E, H, K, N) PPG. (C, F, I, L, O) Normal-tension glaucoma. Note: LC thickness gradually declined with glaucoma severity.

The coefficient of variation for avgLCT was 5.1%. An analysis of inter-observer variation between two investigators revealed a coefficient of variation of 4.9%. Representative B-scan images of normal eyes, eyes with PPG, and eyes with NTG are shown in Fig 5A and 5C. LC thickness gradually declined with the severity of glaucoma. Fig 5M and 5O shows representative LC map results, with the validated area shown in color. The LC map also showed that LC thickness declined with the severity of glaucoma.

**Fig 6:**
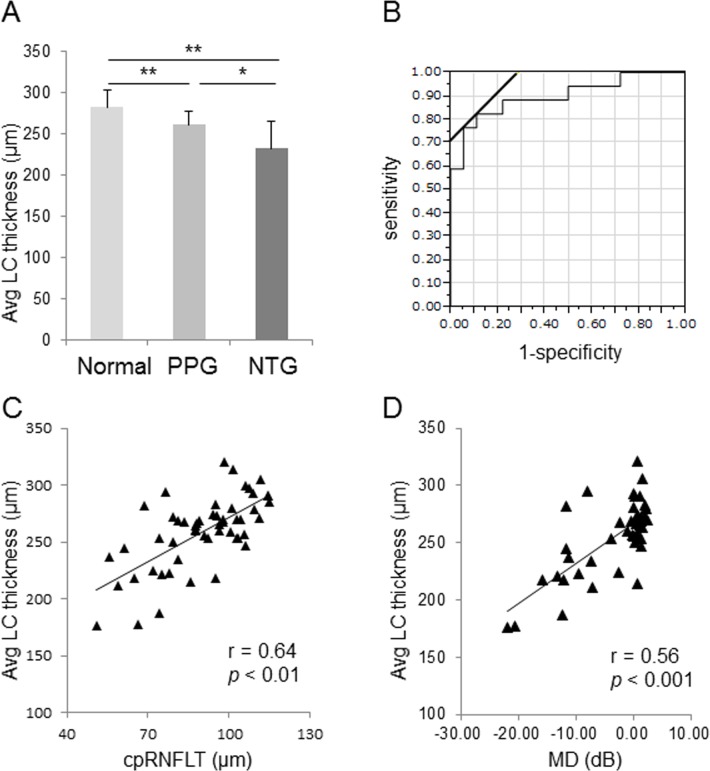
Correlation between average lamina cribrosa (LC) thickness and circumpapillary retinal nerve fiber layer thickness (cpRNFLT). (A) Bar graph indicating LC thickness in different stages. Note: there were significant differences between these groups (Kruskal-Wallis test followed by Steel-Dwass test). *: P<0.05, **: P<0.01. (B) ROC curve. The area under the ROC curve was 0.9, with a cutoff value of 260.4 μm (sensitivity: 0.83; specificity: 0.89). (C) Scatter plot of cpRNFLT against average LC thickness (avgLCT) in the entire group (N = 54). (D) Scatter plot of MD against avgLCT in the entire group (N = 54). Note: the correlation coefficient of avgLCT and cpRNFLT was 0.64 (p < 0.01) and the correlation coefficient of avgLCT and HFA MD was 0.56 (p < 0.001).

AvgLCT was 282.6 ± 20.6 μm in the normal eyes, 261.4 ± 15.8 μm in the PPG eyes, and 232.6 ± 33.3 μm in the NTG eyes. The differences between these groups were significant (Normal vs. PPG: p < 0.01, Normal vs. NTG: p < 0.01, PPG vs. NTG: p < 0.05). ROC curves are shown in Fig 6B. The area under the ROC curve was 0.9, with a cutoff value of 260.4 μm with the Youden index (sensitivity: 0.83; specificity: 0.89). The correlation coefficient between avgLCT and cpRNFLT was 0.64 (p < 0.01, Fig 6C) and the correlation coefficient between avgLCT and HFA MD was 0.56 (p < 0.001, Fig 6D) in the patients overall. A partial correlation coefficient analysis, corrected for age, revealed that the correlation coefficient for avgLCT and cpRNFLT was 0.59, and that for avgLCT and MD it was 0.67.

## Discussion

In this study, we developed new software to measure the average thickness of the LC within a validated area of an SS-OCT disc scan image. We then investigated the usefulness of this new measurement parameter in 54 eyes (Normal: 18, PPG: 18, and NTG: 18). Our software enabled us to synchronize reconstructed B- and *en-face* images, and to visualize these images simultaneously, by displaying both a B-scan image and corresponding *en-face* images from various depths on the same screen. In the *en-face* images, we marked the top and bottom borders of the LC according to points where the visible pores of the LC first became visible and ceased being visible, respectively. We found that our measurements of LC thickness were highly reproducible (CV = 5.1%), and that avgLCT and cpRNFLT were closely correlated (0.64, p < 0.01). We were thus able to validate the accuracy of our new measurement parameter. Therefore, our newly developed method for evaluating the LC can be considered reliable, and promises to become a valuable part of clinical assessments of NTG in the future.

Measurements of different aspects of optic nerve cupping with the Heidelberg retinal tomography 2 device [30,31] or with stereo fundus photography have previously been shown to be valuable indicators of glaucoma severity [32]. Recent research has also shown that OCT can be used to quantify the formation of the cup and rim by setting Bruch’s membrane opening (BMO) as the baseline [33]. Additionally, it has been found that the anterior border of the LC is easier to measure than the posterior border, and that posterior displacement of the LC surface may occur in glaucoma, a finding that provides the basis for future *in vivo* human studies [24]. These findings have prompted the exploration of a wide variety of new approaches to the evaluation of optic disc parameters, but all of these have used indirect methods.

The critical problem in measuring LC thickness is accurately identifying the outer borders of the LC. EDI-OCT with maximum-intensity projection has been shown to improve the visibility of the anterior and posterior borders of the LC [34], but EDI-OCT has well-known limitations in measuring the LC in areas beneath the neuroretinal rim. SS-OCT uses a longer wavelength laser as a light source that enables deeper penetration than EDI-OCT, and thus may have a superior ability to detect the posterior border of the LC. Lopilly Park et al. showed that these two OCT technologies had similar detection rates for the posterior border of the LC (EDI-OCT: 75%, SS-OCT: 81%) [35], but found that SS-OCT measurements of the posterior border of the LC were deeper (306 μm) than EDI-OCT measurements (267 μm). Furthermore, as SS-OCT uses a central wavelength of 1,050 nm that is invisible to the human eye, patients can maintain a relatively stable fixation during imaging, improving the quality of the images. SS-OCT thus has technical characteristics that can improve noise reduction and the quality of merged images. In this study, we reconstructed *en-face* images of the LC from OCT disc volume data, using a motion-averaging method in the *z* direction. This method, based on SS-OCT, has allowed us to achieve the most accurate visualization of the outer-border thickness of the LC reported so far.

Previous reports on EDI-OCT-measured LC thickness have been inconsistent. Park et al. found that LC thickness was 348.14 ± 23.41 μm in normal subjects, 237.82 ± 40.32 μm in POAG patients, and 175.11 ± 22.60 μm in NTG patients, in a study using SD-OCT (Spectralis, Heidelberg) [36]. Inoue et al. found that the thickness of the LC in glaucoma patients was 190.5 ± 52.7 μm, in a study also using SD-OCT (3D OCT-1000, TOPCON Corporation) [26]. Here, we found that average LC thickness was 232.6 ± 33.3 μm in a group of NTG patients. The discrepancies between these reports may arise from differing definitions of the outer border of the LC and different measurement locations. Previous reports defined the outer border of the LC as the point where the OCT signal disappeared, and measured the average of 3 horizontal lines in the optic nerve head. In this study, we defined the outer border of the LC as the point where the LC pores disappeared, and determined this point in 12 radial B-scan images. Recently, volume-rendered 3D images of the optic disc have shown that the anterior border of the LC can include a central horizontal ridge [23] or focal concavity, can be flat or sloped, and can be W- or U-shaped [27]. This anatomical variety suggests that LC thickness measurements obtained from only a small area may be inaccurate. Our method of defining the outer border of the LC should therefore provide a more accurate assessment of LC thickness. Thus, although the true average thickness of the LC remains unclear, we were able to obtain highly reproducible measurements that were closely correlated to glaucoma severity, indicating that our method may be a promising new way of evaluating glaucomatous damage.

In this study, we measured the thickness of the LC only in areas of the scanned image that had been established as reliable. The limitations of OCT technology produce shadows beneath retinal vessels, and the optic disc is an area particularly rich in vessels. The resulting high concentration of shadows can thus affect the accurate assessment of the LC. Indeed, we found that OCT shadows also risked affecting the manual process of segmenting the LC, impeding the accurate segmentation of the posterior border and flank of the LC. Thus, we manually defined an area of the scanned image that could be reliably segmented and measured LC thickness only in this area. We found that the correlation coefficient between LC thickness and the severity of glaucomatous damage was stronger when only the reliably measurable area was included in the analysis. This result suggests that evaluations of LC thickness would be improved by including only areas of the OCT scan where the LC can be reliably measured. In the near future, enhancement techniques such as adaptive compensation [37] may enable clearer visualization of the LC surface beneath the vessels or thick sections of the neuronal rim, thereby increasing the accuracy of measurements of LC thickness.

The goal of this study was to evaluate new software for the analysis of SS-OCT scans. This study included only a small study population, composed of subjects from a single ethnic group. Additionally, it remains unclear whether factors such as age and myopia affect LC thickness. Regarding the influence of tilted disc in measuring LC thickness, our method would overestimate the thickness of the LC. For example, if the lamina were tilted by 5 degrees, the measured thickness would increase by around 8%. However, to minimize potential bias from these factors, we matched age and refractive error between the groups. Finally, the version of the software used in this study required approximately 40 minutes for the operator to complete the entire process and form a 3D image. Most of this time was used in the manual marking process. If the border of the LC was flat, it took less than 15 minutes to complete this process, but if the border was curved, the process was lengthier. Hence, the version of our method described here is not suitable for normal clinical use. As a next step, we are planning to develop a new version of the software that has less need for manual marking, is quicker to use, and can automatically segment the LC borders. However, the version of the software described here was able to produce a detailed 3D image of the LC, demonstrating its suitability for glaucoma research.

## Conclusions

In conclusion, this is the first report on a successful method to segment the LC *in vivo* in 3D, and to measure the average thickness of the LC. Although the effect of retinal vessel shadows in the optic nerve head on assessment of the LC was not entirely eliminated, we found that our measurements of avgLCT were significantly correlated to glaucoma severity. Therefore, our new method of measuring LC thickness with SS-OCT can be considered a reliable, useful and accurate parameter for the evaluation of LC structure, and would enhance research into the pathology of NTG.
